# Hierarchical control of differential steering for four-in-wheel-motor electric vehicle

**DOI:** 10.1371/journal.pone.0285485

**Published:** 2023-06-09

**Authors:** Jie Tian, Mingfei Yang

**Affiliations:** College of Automobile and Traffic Engineering, Nanjing Forestry University, Nanjing, China; Zonguldak Bülent Ecevit University: Zonguldak Bulent Ecevit Universitesi, TURKEY

## Abstract

The purpose of this paper is to study the control of differential steering for four-in-wheel-motor electric vehicles. The so-called differential steering means that the front wheel steering is realized through the differential driving torque between the left and right front wheels. With the consideration of tire friction circle, a hierarchical control method is proposed to realize the differential steering and the constant longitudinal speed simultaneously. Firstly, the dynamic models of the front wheel differential steering vehicle, the front wheel differential steering system and the reference vehicle are established. Secondly, the hierarchical controller is designed. The upper controller is to obtain the resultant forces and resultant torque required by the front wheel differential steering vehicle tracking the reference model through the sliding mode controller. In the middle controller, the minimum tire load ratio is selected as the objective function. Combined with the constraints, the resultant forces and resultant torque are decomposed into the longitudinal and lateral forces of four wheels by the quadratic programming method. The lower controller provides the required longitudinal forces and tire sideslip angles for the front wheel differential steering vehicle model through the tire inverse model and the longitudinal force superposition scheme. Simulation results show that the hierarchical controller can guarantee the vehicle to track the reference model well on both of the high and low adhesion coefficient road with all of the tire load ratios smaller than 1. It can be drawn that the control strategy proposed in this paper is effective.

## Introduction

Electric vehicles driven by hub motors have become one of the research hotspots because of their advantages of energy saving, environmental protection, simple structure and easy to realize the integrated control [1, 2]. For these multi-actuated electric vehicles, a lot of research have been done, such as the fault-tolerant control [3], the chassis coordinated control [4, 5], the integrated vehicle-following control [6–8], the energy management strategy [9–11], the battery state-of-charge (SOC) estimation [12–14], the driving modes [15–17], the strength analysis of key components [18, 19] and so on. In addition, their appearances also make the differential steering possible, which can be used not only to assist the driver to turn the vehicle [20–23], but also as a backup system [24–28] and even the only steering system of the vehicle [2, 29, 30], which can further simplify the structure of the vehicle. Among them, the literatures [2, 25–30] are most related to this study.

To realize the yaw control by the differential steering, an integral sliding mode control (ISMC) was proposed for in-wheel-motor (IWMD) electric vehicles. In order to eliminate the chattering effect, an adaptive super-twisting control approach was proposed to deal with the disturbances with unknown boundaries. The simulation results verified the effectiveness of the proposed controller [25]. The dynamic model differential steering vehicle (DSV) was established. The sliding mode controller was designed for the DSV to track the yaw rate and sideslip angle of the traditional front-wheel steering vehicle with neutral steering characteristics. By comparing with the designed controller for the skidding steering vehicle (SSV), the simulation results proved the possibility of the differential steering in case of steering failure [26]. In order to realize the four-wheel differential steering function of an in-wheel motor (IWM) driven electric vehicle (EV), the decoupling and fractional PID controllers were designed for the four-wheel steering vehicle (4WSV) to obtain the front and rear wheel steering angles. Then the sliding mode controller (SMC) and torque distributor were designed to control the four-wheel differential steering vehicle (4WDSV) to have the same yaw rate as the 4WSV. And the simulation results verified the effectiveness of the proposed controllers [27].

The *H*_∞_ observer-based schema was employed for IM EVs differential steering control with rollover consideration to achieve the yaw rate tracking by differential steering and minimize the rollover risk. And the simulation results proved the effectiveness of the proposed controller [28]. A differential steering fully realized by the drive torque differences was presented. A planar and non-linear vehicle model was built, a full state feedback control system based on linear quadratic regulator (LQR) principle was developed for higher speeds to track the reference sideslip angle and yaw rate, and a simple PI angle tracking controller were proposed for lower speeds to track the reference steering input. Various simulation experiments demonstrated that the differential steering system (DSS) can be a useful alternative to conventional steering [29].

Using the robust *H*_∞_ controller to control the forward speed and yaw rate, a differential steering by the input torque of the four wheels was achieved for an independent four-wheel drive electric vehicle (EV). By comparing with the all-wheel drive (AWD), the rear wheel drive (RWD), and the front wheel drive (FWD), the effectiveness of the robust controller was proved, which showed that the differential steering can effectively maneuver the vehicle under different driving conditions [30]. Considering the strong coupling between mechanical and control components of the differential steering vehicle, three design objectives and three constraints were defined for its dynamic, steady and low-speed steering performance, and optimization was carried out through multi-objective genetic algorithm based on iteratively updated neural network-based response surface metamodels. The results indicated that vehicles equipped with the differential steering can provide convincing steering performance [2].

To sum up, all of the above documents have realized the tracking of the reference yaw rate and even the reference centroid sideslip angle of the differential steering vehicle through the design of various controllers, and some even considered the vehicle rollover problem. In a word, the feasibility of differential steering has been confirmed to a certain extent. However, the above studies almost assumed that the vehicle speed was constant during the steering, and does not consider the nonlinearity of the tire, let alone the friction circle of the tire. That is to say, when the vehicle is driving at a constant speed and turning at the same time, the tires may not meet the two needs in the reality. In addition, if the load on the one side front wheel decreases sharply during the steering, it will also cause the wheel to be unable to generate enough driving force, which will lead to the failure of differential steering. In order to ensure that differential steering can be used on real vehicles in the future, it is necessary to consider the friction circle of the tire to conduct in-depth research on the effectiveness of differential steering.

The contribution of this paper lies in: (1) Under the condition of considering the tire attachment circle, the front wheel differential steering is realized while ensuring the constant vehicle speed; (2) In order to track the longitudinal speed, the lateral speed and the yaw rate of the reference model, a sliding mode controller is designed to obtain the longitudinal, lateral resultant forces, and the resultant moments required by the front wheel differential steering vehicle (FWDSV); (3) Taking the minimum tire load ratio as the objective function, and the restrictions of tire attachment circle, wheel motor driving torque, resultant force and resultant moment as the constraints, the longitudinal and lateral forces required by each wheel are obtained through quadratic programming; (4) On this basis, through the tire inverse model and the superposition scheme, the longitudinal and lateral forces required by the four wheels are converted into the input parameters required by the FWDSV to complete the closed-loop control.

The rest of this paper is organized as follows the FWDSV, the front wheel differential steering system (FWDSS) and the reference model are described in Section II. In Section III the hierarchical control system including the upper, middle and low controllers is designed. Simulations and discussion are carried out in Section IV. Final conclusions are drawn in the Section V.

## Dynamic models

In this section, three dynamic models are established, including the FWDSV model, the FWDSS model and the reference model. The FWDSV model is used to generate the actual longitudinal speed, lateral speed and yaw rate according to the longitudinal and lateral forces of the four wheels. The FWDSS model is used to obtain the actual longitudinal forces required by the front wheels according to the front wheel steering angle generated by the differential steering vehicle. The reference model is used to generate the desired longitudinal velocity, lateral velocity and yaw rate according to the given front wheel steering angle.

### Front wheel differential steering vehicle model

**Fig 1:**
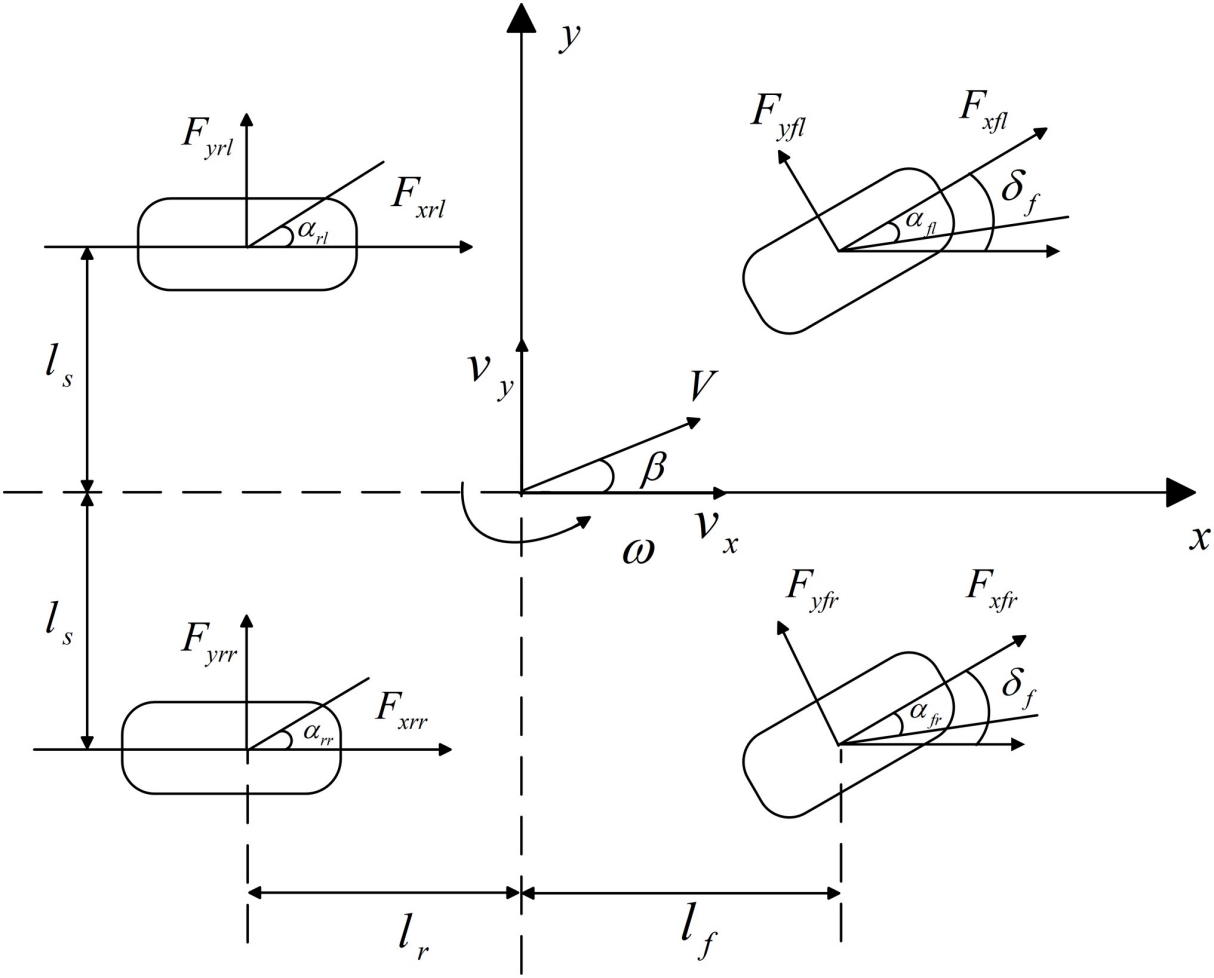
Vehicle plane dynamic model.

The four-in-wheel-motor (4IWM) EV studied in this paper adopts the differential steering system to realize the normal front wheel steering function. In order to simplify the dynamic model, the pitch motion, roll motion and parameter uncertainty of the vehicle are ignored. The resulting vehicle plane dynamic model is shown in Fig 1.

The vehicle dynamic equation obtained from Fig 1 is as follows:

$$\left\{\begin{array}{l} m({\dot{v}}_{x}-v_{y}\omega )=F_{xd}\\ m({\dot{v}}_{y}+v_{x}\omega )=F_{yd}\\ I_{Z}\dot{\omega }=M_{d} \end{array}\right.,$$ (1)

$$F_{xd}=F_{xfl}+F_{xfr}+F_{xrl}+F_{xrr},\;F_{yd}=F_{yfl}+F_{yfr}+F_{yrl}+F_{yrr},\;M_{d}=l_{f}(F_{yfl}+F_{yfr})-l_{r}(F_{yrl}+F_{yrr})+l_{s}(F_{xfr-}F_{xfl}),$$

where, *m* is the total mass of the vehicle, *v*_*x*_ and *v*_*y*_ are the longitudinal and lateral velocities respectively, ${\dot{v}}_{x}$ and ${\dot{v}}_{y}$ are the longitudinal and lateral accelerations respectively, *ω* is the yaw rate, *F*_*xd*_, *F*_*yd*_ and *M*_*d*_ are the longitudinal resultant force, lateral resultant force and resultant torque of the FWDSV model, *F*_*xij*_ (*i* = *f*, *r*, *j* = *l*, *r*) represents the longitudinal forces of four wheels, *F*_*yij*_ (*i* = *f*, *r*, *j* = *l*, *r*) represents the lateral forces of four wheels, *I*_*z*_ is the yaw moment of inertia, *l*_*f*_ and *l*_*r*_ are respectively the distances between the vehicle centroid and the front and rear axles, *l*_*s*_ is half of the wheel-track.

**Remark 1**. *F*_*xd*_, *F*_*yd*_ and *M*_*d*_ of Eq (1) can be obtained by the upper controller, which makes the FWDSV model track the longitudinal velocity, the lateral velocity and the yaw rate of the reference model.

Considering that the wheel steering angle is small and only the front wheels turn, then

$$F_{yij}=k_{i}a_{ij}\left(i=f,r,j=l,r\right),$$ (2)

$$\left\{\begin{array}{l} \alpha_{f}=\alpha_{fl}=\alpha_{fr}=-\beta -\frac{l_{f}\omega }{v_{x}}+\delta_{f}\\ \alpha_{r}=\alpha_{rl}=\alpha_{rr}=-\beta +\frac{l_{r}\omega }{v_{x}} \end{array}\right.,$$ (3)

where, *k*_*i*_(*i* = *f*, *r*) is the front/rear wheel cornering stiffness, *α*_*ij*_(*i* = *f*, *r*, *j* = *l*, *r*) is the tire sideslip angle of front/rear left/right wheel, *a*_*i*_(*i* = *f*, *r*) is the front/rear tire sideslip angle, *β* is the sideslip angle, *δ*_*f*_ is the front wheel steering angle.

Substituting Eqs (2) and (3) into Eq (1), then Eq (1) can be rewritten as:

$$\left\{\begin{array}{l} m({\dot{v}}_{x}-v_{y}\omega )=F_{xfl}+F_{xfr}+F_{xrl}+F_{xrr}\\ m({\dot{v}}_{y}+v_{x}\omega )=k_{f}\alpha_{fl}+k_{f}\alpha_{fr}+k_{r}\alpha_{rl}+k_{r}\alpha_{rr}\\ I_{Z}\dot{\omega }=l_{f}k_{f}(\alpha_{fl}+\alpha_{fr})-l_{r}k_{r}(\alpha_{rl}+\alpha_{rr})+l_{s}(F_{xfr}-F_{xfl}) \end{array}\right..$$ (4)

**Remark 2**. When *F*_*xij*_ (*i* = *f*, *r*, *j* = *l*, *r*) and *α*_*ij*_(*i* = *f*, *r*, *j* = *l*, *r*) are obtained, *v*_*x*_, *v*_*y*_ and *ω* of the FWDSV model can be obtained. Therefore, it is necessary for the middle controller to decompose *F*_*xd*_, *F*_*yd*_ and *M*_*d*_ into *F*_*xij*_ (*i* = *f*, *r*, *j* = *l*, *r*) and *F*_*yij*_ (*i* = *f*, *r*, *j* = *l*, *r*) of each wheel, and then further convert *F*_*yij*_ (*i* = *f*, *r*, *j* = *l*, *r*) into the sideslip angles of each wheel and the longitudinal forces of the front wheels by the lower controller.

And the corresponding vertical load of each wheel can be expressed as:

$$\left\{\begin{array}{l} F_{zfl}=\frac{m}{l_{f}+l_{r}}\left(\frac{l_{r}g}{2}-\frac{a_{x}h}{2}-\frac{a_{y}hl_{r}}{2l_{s}}\right)\\ F_{zfr}=\frac{m}{l_{f}+l_{r}}\left(\frac{l_{r}g}{2}-\frac{a_{x}h}{2}+\frac{a_{y}hl_{r}}{2l_{s}}\right)\\ F_{zrl}=\frac{m}{l_{f}+l_{r}}\left(\frac{l_{f}g}{2}+\frac{a_{x}h}{2}-\frac{a_{y}hl_{f}}{2l_{s}}\right)\\ F_{zrr}=\frac{m}{l_{f}+l_{r}}\left(\frac{l_{f}g}{2}+\frac{a_{x}h}{2}+\frac{a_{y}hl_{f}}{2l_{s}}\right) \end{array}\right.,$$ (5)

where, *h* is the height from the vehicle centroid to the ground, *g* is the gravitational acceleration.

### Front wheel differential steering system model

**Fig 2:**
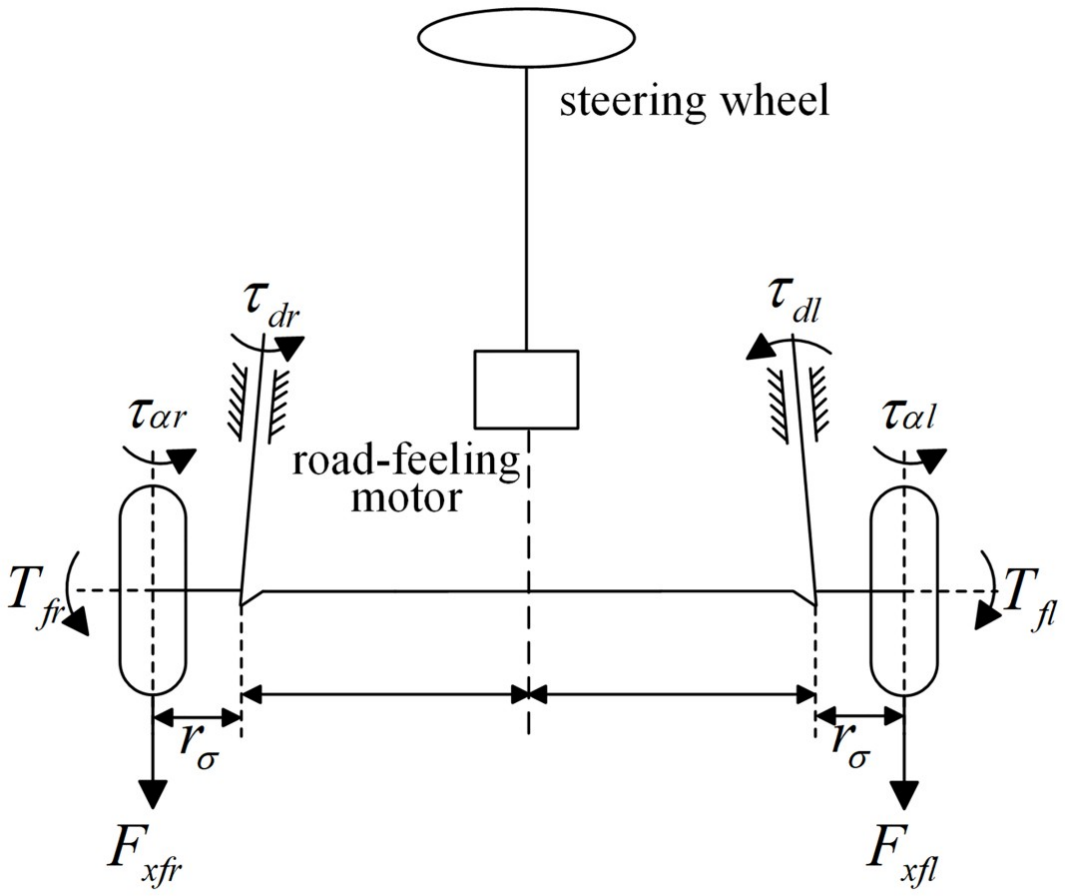
Front wheel differential steering system.

Fig 2 shows the structure of front wheel differential steering system, where *T*_*fl*_ and *T*_*fr*_ are the driving torques of the left and right front wheels.

The dynamic equation of FWDSS model established by Fig 2 is as follows:

$$\left\{\begin{array}{l} J_{e}{\ddot{\delta }}_{f}+b_{e}{\dot{\delta }}_{f}=\Delta M+\tau_{a}-\tau_{f}\\ \tau_{\alpha }=\tau_{\alpha r}+\tau_{\alpha l}=2k_{f}\alpha_{f}l^{2}/3\\ \Delta M=\tau_{dr}-\tau_{dl}=(F_{xfr}-F_{xfl})r_{\sigma }\\ \tau_{dl}=F_{xf\text{l}}\cdot r_{\sigma }\\ \tau_{dr}=F_{xfr}\cdot r_{\sigma } \end{array}\right.,$$ (6)

where, *J*_*e*_ is the equivalent inertia moment of the steering system, *b*_*e*_ is the equivalent steering damping, Δ*M* is the difference between the moments of the two front wheels around their kingpins, *τ*_*αl*_ and *τ*_*αr*_ are the aligning moments of the left and right front wheels, *τ*_*α*_ is the total aligning moment of the front wheels, *τ*_*f*_ is the friction moment of the steering system, *l* is the half width of the tire grounding, *r*_*σ*_ is the scrub radius.

Because the dry friction torque of vehicle steering system, *τ*_*f*_, is small and difficult to measure, its influence can be ignored. At the same time, because the value of ${\ddot{\delta }}_{f}$ is small, it can also be ignored. Due to the longitudinal force of the front wheel, *F*_*xf*l_ = *T*_*fj*_/*R*_*c*_, (*j* = *l*, *r*), where *R*_*c*_ is the wheel radius, we can get:

$$\Delta M=(T_{fr}-T_{fl})\frac{r_{\sigma }}{R_{c}}=\frac{r_{\sigma }}{R_{c}}\Delta T,$$ (7)

where, Δ*T* is the differential driving torque between the left and right front wheels.

Substituting Eq (7) into Eq (6), then Eq (6) can be rewritten as:

$${\dot{\delta }}_{f}=\frac{l^{2}k_{f}}{3b_{e}}\delta_{f}-\frac{l^{2}k_{f}}{3b_{e}v_{x}}v_{y}-\frac{l^{2}k_{f}l_{f}}{3b_{e}v_{x}}\omega +\frac{r_{\sigma }}{b_{e}R_{c}}\Delta T.$$ (8)

### Reference model

In this paper the typical two degree of freedom linear vehicle model is selected as the reference model to provide the ideal lateral velocity *v*_*yd*_, and yaw rate *ω*_*d*_ under the premise of given front wheel steering angle *δ*_*fd*_, and longitudinal velocity *v*_*xd*_.

Set the state space variable as *x*_*d*_(*t*) = [*β*_*d*_, *ω*_*d*_], and the system input as the front wheel steering angle *δ*_*fd*_, that is, *u*_*d*_(*t*) = [*δ*_*fd*_], the corresponding state equation of the reference model is:

$$\left\{\begin{array}{l} {\dot{x}}_{d}=A_{d}x_{d}+B_{d}u_{d}\\ y_{d}=C_{d}x_{d}+D_{d}u_{d} \end{array}\right.,$$ (9)

$$A_{d}=\left(\begin{array}{cc} \frac{\left(k_{f}+k_{r}\right)}{mv_{xd}} & \frac{\left(l_{f}k_{f}-l_{r}k_{r}\right)}{mv_{xd}{}^{2}}-1\\ \frac{\left(l_{f}k_{f}-l_{r}k_{r}\right)}{I_{z}} & \frac{\left(l_{f}^{2}k_{f}+l_{r}^{2}k_{r}\right)}{I_{z}v_{xd}} \end{array}\right),\;B_{d}=\left(\begin{array}{c} -\frac{k_{f}}{mv_{xd}}\\ -\frac{l_{f}k_{f}}{I_{z}} \end{array}\right),\;C_{d}=\left(\begin{array}{cc} 1 & 0\\ 0 & 1 \end{array}\right),\;D_{d}=\left(\begin{array}{c} 0\\ 0 \end{array}\right).$$

According to the relationship between the sideslip angle and the lateral velocity, the ideal lateral velocity can be obtained as:

$$v_{yd}=v_{xd}\cdot \text{tan}\;\beta_{d}.$$ (10)

## Design of hierarchical control system

**Fig 3:**
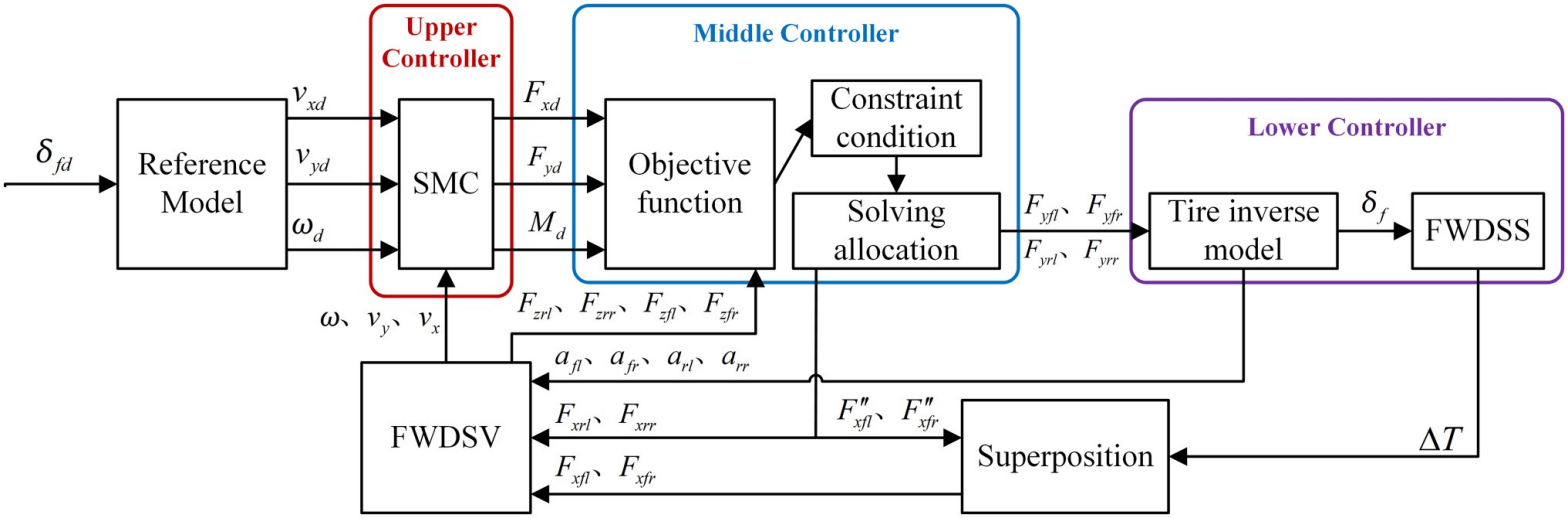
Hierarchical control system.

For the 4IWM EV with the differential steering, this paper designs a hierarchical control system as shown in Fig 3.

It can be seen from Fig 3 that the reference model outputs the ideal longitudinal velocity *v*_*xd*_, the lateral velocity *v*_*yd*_ and the yaw rate *ω*_*d*_ under the given front wheel steering angle *δ*_*fd*_. The upper controller calculates the expected longitudinal resultant force *F*_*xd*_, lateral resultant force *F*_*yd*_ and resultant torque *M*_*d*_ required by the FWDSV model to track the reference model based on the actual longitudinal velocity *v*_*x*_, lateral velocity *v*_*y*_ and yaw rate *ω* output from the FWDSV model. According to the objective function and constraint condition, the middle controller distributes the resultant forces and torque, *F*_*xd*_, *F*_*yd*_ and *M*_*d*_, into the longitudinal and lateral forces of the four wheels, i.e., *F*_*yij*_(*i* = *f*, *r*, *j* = *l*, *r*), ${F^{\prime \prime }}_{xfj}\left(j=l,r\right)$, *F*_*xrj*_(*j* = *l*, *r*), by the optimization solution.

The lower controller converts the lateral forces of four wheels *F*_*yij*_(*i* = *f*, *r*, *j* = *l*, *r*) into the front wheel steering angle *δ*_*f*_ and the tire sideslip angles of four wheels *α*_*ij*_(*i* = *f*, *r*, *j* = *l*, *r*) through the tire inverse model. Then the front wheel steering angle *δ*_*f*_ is input to the FWDSS model to obtain the differential driving torque between the left and right front wheels Δ*T*, which is finally transformed to the longitudinal forces of the rear wheels *F*_*xfj*_(*j* = *l*, *r*). Finally, the longitudinal forces of the four wheels *F*_*xij*_(*i* = *f*, *r*, *j* = *l*, *r*) and the sideslip angles of four wheels *α*_*ij*_(*i* = *f*, *r*, *j* = *l*, *r*) are also provided to the FWDSV model. In this way, the control system forms a closed loop.

### Design of upper controller

Sliding mode variable structure control is a control method with strong robustness, which has the advantages of fast response and insensitive to the external changes and disturbances. The control of exponential reaching law can also effectively reduce the system jitter [29]. Therefore, it is used to design of the upper controller.

According to the FWDSV model and the reference model established above, the tracking errors of the actual and ideal longitudinal velocities, lateral velocities and yaw rates are set as *e*_1_, *e*_2_, *e*_3_, respectively.


$$\left\{\begin{array}{l} e_{1}=v_{x}-v_{xd}\\ e_{2}=v_{y}-v_{yd}\\ e_{3}=\omega -\omega_{d} \end{array}\right..$$ (11)

Different sliding surfaces are selected for the control of the longitudinal velocity, the lateral velocity and the yaw rate.

$$\left\{\begin{array}{l} s_{1}=c_{1}e_{1}\\ s_{2}=c_{2}e_{2}\\ s_{3}=c_{3}e_{3} \end{array}\right.,$$ (12)

where, *c*_1_, *c*_2_, *c*_3_ are the controller parameters that must meet the Hurwitz condition, and their values are greater than zero.

The exponential approach law is adopted to reduce the system chattering. The expression is as follows:

$$\left\{\begin{array}{l} {\dot{s}}_{1}=-\varepsilon_{1}\;\text{sgn}\;s_{1}-k_{1}s_{1}\\ {\dot{s}}_{2}=-\varepsilon_{2}\;\text{sgn}\;s_{2}-k_{2}s_{2}\\ {\dot{s}}_{3}=-\varepsilon_{3}\;\text{sgn}\;s_{3}-k_{3}s_{3} \end{array}\right.,$$ (13)

where, *ε*_1_, *ε*_2_, *ε*_3_, *k*_1_, *k*_2_ and *k*_3_ are the controller parameters, and their values are greater than zero.

In the process of sliding mode switching, by increasing *k* and decreasing *ε* at the same time, the system will be less affected and the transition will be more stable. From the accessibility of a sliding mode controller, $s=\dot{s}=0$ must be satisfied, then

$$\left\{\begin{array}{l} {\dot{v}}_{x}=\frac{-\varepsilon_{1}\;\text{sgn}\;s_{1}-k_{1}s_{1}}{c_{1}}+{\dot{v}}_{xd}\\ {\dot{v}}_{y}=\frac{-\varepsilon_{2}\;\text{sgn}\;s_{2}-k_{2}s_{2}}{c_{2}}+{\dot{v}}_{yd}\\ \dot{\omega }=\frac{-\varepsilon_{3}\;\text{sgn}\;s_{3}-k_{3}s_{3}}{c_{3}}+{\dot{\omega }}_{d} \end{array}\right..$$ (14)

In order to reduce the chattering of system, the sgn *s* in Eq (13) is replaced by a saturation function *sat*(*s*_*n*_/Δ_*n*_), where *n* = 1, 2, 3. And

$$sat\left(s_{n}/\Delta_{n}\right)=\left\{\begin{array}{ll} s_{n}/\Delta_{n} & \left|s_{n}/\Delta_{n}\right|\;\leq 1\\ \text{sgn}\left(s_{n}/\Delta_{n}\right) & \left|s_{n}/\Delta_{n}\right|\;>\;1 \end{array}\right.,$$ (15)

where, Δ_*n*_ is the boundary layer. The larger its value is, the less chattering will be. However, at the same time the approaching speed will slow down.

By combining Eqs (1) and (14), the output of the sliding mode controller can be solved, that is, the required resultant forces and torque.


$$\left\{\begin{array}{l} F_{xd}=m\left[\frac{-\varepsilon_{1}sat\left(s_{1}/\Delta_{1}\right)-k_{1}s_{1}}{c_{1}}+{\dot{v}}_{xd}-v_{y}\omega \right]\\ F_{yd}=m\left[\frac{-\varepsilon_{2}sat\left(s_{2}/\Delta_{2}\right)-k_{2}s_{2}}{c_{2}}+{\dot{v}}_{yd}+v_{x}\omega \right]\\ M_{d}=I_{z}\left[\frac{-\varepsilon_{3}sat\left(s_{3}/\Delta_{3}\right)-k_{3}s_{3}}{c_{3}}+{\dot{\omega }}_{d}\right] \end{array}\right..$$ (16)

### Design of middle controller

In this section, the expected resultant forces and torque obtained by the upper controller should be optimally decomposed into the longitudinal and transverse forces of four wheels according to the objective function. Therefore, the objective function, constraint conditions and optimization solution will be researched in the following.

#### Objective function

Considering that the tire load ratio represents the stability margin of the tire, and the lower the tire load ratio, the greater the stability margin. Therefore, in this paper the minimum tire load ratio is selected as the optimization objective. The so-called tire load ratio refers to the ratio of the adhesion force between the single wheel and the ground to the maximum adhesion force under the current road conditions. And the objective function can be expressed as [30]:

$$\text{min}\;J=\sum_{i=l}^{r}\sum_{i=f}^{r}\frac{F_{xij}^{2}+F_{yij}^{2}}{\mu_{}^{2}F_{zij}^{2}},$$ (17)

where, *μ* is the adhesion coefficient of the road where the tires are located.

The value range of tire load ratio is [0,1]. The smaller the tire load ratio is, the more force the tire can output and the greater the stability margin is. In other words, the closer the tire load ratio is to 1, the worse the controllability of the vehicle is. When the tire load ratio equals to 1, it indicates that the tire has reached the limit of its adhesion capacity. If the vehicle is disturbed at this time, it will lose stability because the tire has no residual force to respond to this disturbance.

#### Constraint conditions

During driving, the 4WIM EV will be affected and limited by the tire friction circle, the maximum driving force of the in-wheel-motor, the resultant forces and resultant torque of the vehicle, therefore it is necessary to define some constraints on the solution of the objective function.

*Constraint of tire friction circle*. Generally, the tire adhesion limit of each wheel shall be regarded as a constraint condition, which can be described by the tire friction circle. That is to say, the following expression shall be met between the tire longitudinal and lateral forces, and vertical load.


$$F_{xij}{}^{2}+F_{yij}{}^{2}\leq {\left(\mu F_{z}{}_{ij}\right)}^{2},\left(i=f,r,j=l,r\right).$$ (18)

**Fig 4:**
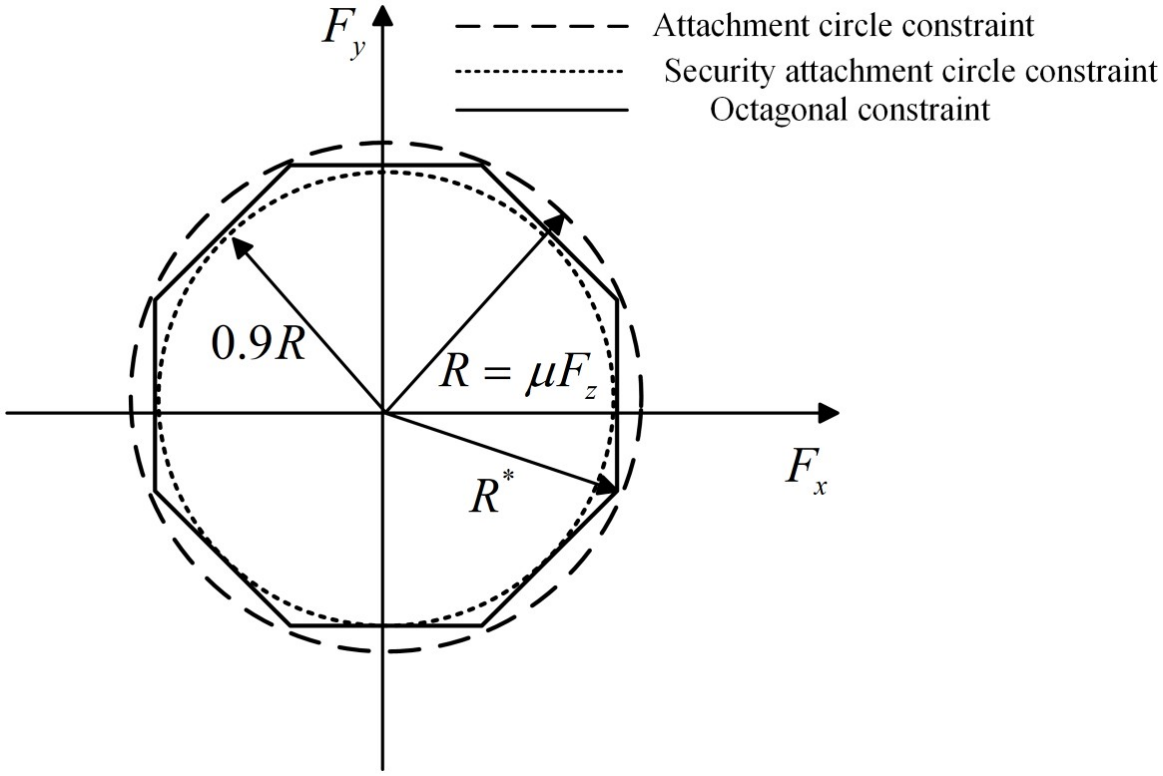
Linear simplified model of tire friction circle.

In this paper, the nonlinear tire friction circle constraint is simplified to a linear polygon constraint. In order to ensure that the longitudinal and lateral forces of each wheel do not exceed the friction circle, a safe friction circle is set up, that is, multiplying the friction circle in Eq (18) by a safety factor of 0.9, and then circumscribing an octagon on the safe friction circle to obtain the required linear constraint, as shown in Fig 4.

The radius of the attached circle in Fig 4 is *μF*_*z*_, and the circumscribed circle radius of the octagon is calculated as follows:

$$R^{*}=0.9R\cdot \text{sec}\;22.5^{{}^{\circ}}\approx 0.97R<R.$$ (19)

Because the radius of the octagonal circumscribed circle is smaller than that of the tire friction circle, each tire force can be limited in the tire friction circle, and its specific linear constraint equation can be described as follows:

$$\left\{\begin{array}{l} -0.9\mu F_{zij}\leq F_{xij}\leq 0.9\mu F_{zij}\\ -0.9\mu F_{zij}\leq F_{yij}\leq 0.9\mu F_{zij}\\ -\sqrt{2}\cdot 0.9\mu F_{zij}\leq F_{xij}+F_{yij}\leq \sqrt{2}\cdot 0.9\mu F_{zij}\\ -\sqrt{2}\cdot 0.9\mu F_{zij}\leq -F_{xij}+F_{yij}\leq \sqrt{2}\cdot 0.9\mu F_{zij} \end{array}\right..$$ (20)

*Constraint of in-wheel-motor maximum driving force*. In this paper the driving force of each wheel is independently controlled by the in-wheel-motor, whose driving torque is limited. Therefore, the longitudinal forces provided by the four tires are limited as follows:

$$-T_{\text{max}}/R_{c}\leq F_{xij}\leq T_{\text{max}}/R_{c},\left(i=f,r,j=l,r\right).$$ (21)

where, *T*_max_ is the maximum torque of the motor, and the value is 600 *N* · *m*.

*Constraint of resultant force and torque*. The expected resultant forces and torque output by the upper controller must be provided by each wheel, so that the constraint of the resultant forces and torque can be obtained as follows:

$$\left\{\begin{array}{l} F_{xd}=F_{xfl}+F_{xfr}+F_{xrl}+F_{xrr}\\ F_{yd}=F_{yfl}+F_{yfr}+F_{yrl}+F_{yrr}\\ M_{d}=l_{f}\left(F_{yfl}+F_{yfr}\right)-l_{r}\left(F_{yrl}+F_{yrr}\right)\text{+}l_{s}\left(F_{xfr}-F_{xfl}\right) \end{array}\right..$$ (22)

#### Optimization solution

Optimization problem is a typical nonlinear control problem. At present, the commonly used optimization algorithms include quadratic programming optimization algorithm, genetic algorithm, particle swarm optimization algorithm [31], etc. Considering that quadratic programming optimization algorithm can be used to solve complex problems or problems with equality and inequality constraints [20], and has high dynamic distribution accuracy and good real-time performance, it is selected to solve the objective function in this paper.

The so-called quadratic programming problem is an optimization problem in which the objective function is a quadratic function and the constraint function is a linear function. Let *x*^*T*^ = (*F*_*xfl*_, *F*_*yfl*_, *F*_*xfr*_, *F*_*yfr*_, *F*_*xrl*_, *F*_*yrl*_, *F*_*xrr*_, *F*_*yrr*_), then the objective function (17) can be rewritten into the standard form of quadratic programming, inequalities (18) and (20) can be written as *Ax* ≤ *b*, Eq (22) can be written as *A*_*eq*_*x* = *b*_*eq*_, and inequality (21) describing the value range of state variables can be rewritten into *lb* ≤ *x* ≤ *ub*, namely:

$${\text{min}}_{x}\;f(x)=\frac{1}{2}x^{T}Hx+f^{T}x,$$ (23)

$$\text{s}.\text{t}.\;\left\{\begin{array}{l} Ax\leq b\\ A_{eq}x=b_{eq}\\ lb\leq x\leq ub \end{array}\right.,$$ (24)

where,

$$H=\frac{2}{\mu^{2}}diag\left(\frac{1}{F_{zfl}{}^{2}},\frac{1}{F_{zfl}{}^{2}},\frac{1}{F_{zfr}{}^{2}},\frac{1}{F_{zfr}{}^{2}},\frac{1}{F_{zrl}{}^{2}},\frac{1}{F_{zrl}{}^{2}},\frac{1}{F_{zrr}{}^{2}},\frac{1}{F_{zrr}{}^{2}}\right),$$

$$f=\left[0;0;0;0;0;0;0;0\right],$$

$$A=\left[\begin{array}{l} 1\;0\;0\;0\;0\;0\;0\;0;\;-1\;0\;0\;0\;0\;0\;0\;0;\;0\;0\;1\;0\;0\;0\;0\;0;\;\\ 0\;0\;-1\;0\;0\;0\;0\;0;\;0\;0\;0\;0\;1\;0\;0\;0;\;0\;0\;0\;0\;-1\;0\;0\;0;\;\\ 0\;0\;0\;0\;0\;0\;1\;0;\;0\;0\;0\;0\;0\;0\;-1\;0;\;0\;1\;0\;0\;0\;0\;0\;0;\;\\ 0\;-1\;0\;0\;0\;0\;0\;0;\;0\;0\;0\;1\;0\;0\;0\;0;\;0\;0\;0\;-1\;0\;0\;0\;0;\\ 0\;0\;0\;0\;0\;1\;0\;0;\;0\;0\;0\;0\;0\;-1\;0\;0;\;0\;0\;0\;0\;0\;0\;0\;1;\;\\ 0\;0\;0\;0\;0\;0\;0\;-1;\;1\;1\;0\;0\;0\;0\;0\;0;\;-1\;-1\;0\;0\;0\;0\;0\;0;\;\\ 0\;0\;1\;1\;0\;0\;0\;0;\;0\;0\;-1\;-1\;0\;0\;0\;0;\;0\;0\;0\;0\;1\;1\;0\;0;\;\\ 0\;0\;0\;0\;-1\;-1\;0\;0;\;0\;0\;0\;0\;0\;0\;1\;1;\;0\;0\;0\;0\;0\;0\;-1\;-1;\;\\ -1\;1\;0\;0\;0\;0\;0\;0;\;1\;-1\;0\;0\;0\;0\;0\;0;\;0\;0\;-1\;1\;0\;0\;0\;0;\;\\ 0\;0\;1\;-1\;0\;0\;0\;0;\;0\;0\;0\;0\;-1\;1\;0\;0;\;0\;0\;0\;0\;1\;-1\;0\;0;\;\\ 0\;0\;0\;0\;0\;0\;-1\;1;\;0\;0\;0\;0\;0\;0\;1\;-1 \end{array}\right],$$

$$b=\left[\begin{array}{l} 0.9\mu F_{zfl};0.9\mu F_{zfl};0.9\mu F_{zfr};0.9\mu F_{zfr};0.9\mu F_{zrl};0.9\mu F_{zrl}\text{;}\\ 0.9\mu F_{zrr};0.9\mu F_{zrr};0.9\mu F_{zfl};0.9\mu F_{zfl};0.9\mu F_{zfr};0.9\mu F_{zfr}\text{;}\\ 0.9\mu F_{zrl};0.9\mu F_{zrl};0.9\mu F_{zrr};0.9\mu F_{zrr};1.3\mu F_{zfl};1.3\mu F_{zfl}\text{;}\\ 1.3\mu F_{zfr};1.3\mu F_{zfr};1.3\mu F_{zrl};1.3\mu F_{zrl};1.3\mu F_{zrr};1.3\mu F_{zrr};\\ 1.3\mu F_{zfl};1.3\mu F_{zfl};1.3\mu F_{zfr};1.3\mu F_{zfr};1.3\mu F_{zrl};1.3\mu F_{zrl}\text{;}\\ 1.3\mu F_{zrr};1.3\mu F_{zrr} \end{array}\right],$$

$$A_{eq}=\left[\begin{array}{cccccccc} 1 & 0 & 1 & 0 & 1 & 0 & 1 & 0\\ 0 & 1 & 0 & 1 & 0 & 1 & 0 & 1\\ -l_{s} & l_{f} & l_{s} & l_{f} & 0 & -l_{r} & 0 & -l_{r} \end{array}\right],$$

$$b_{eq}=\left[F_{xd};F_{yd};M_{d}\right],$$

$$l_{b}=\left[-\frac{T_{\text{max}}}{R_{c}};-\frac{T_{\text{max}}}{R_{c}};-\frac{T_{\text{max}}}{R_{c}};-\frac{T_{\text{max}}}{R_{c}}\right],$$

$$u_{b}=\left[\frac{T_{\text{max}}}{R_{c}};\frac{T_{\text{max}}}{R_{c}};\frac{T_{\text{max}}}{R_{c}};\frac{T_{\text{max}}}{R_{c}}\right].$$

If *H* is a positive semidefinite matrix, then *f (x)* is a convex function. The corresponding quadratic programming is a convex quadratic programming problem. If there is a local optimal solution at this point, then this local optimal solution is the global optimal solution. But this global minimum may not be unique. If *H* is a positive definite matrix, then the problem has a unique global minimum. From the expression of *H*, it is not difficult to draw a conclusion that *H* in this paper is a positive definite matrix. Compiling the s-function in MATLAB/Simulink for Eqs (23) and (24), calling the function quadprog (*H*,*f*,*A*,*b*,*Aeq*,*beq*,*lb*,*ub*,*x*_0_,options) provided by Matlab to solve the optimization problem, and the longitudinal and lateral forces required by each wheel can be calculated.

### Design of lower controller

The purpose of the lower controller is to convert the lateral force of each wheel obtained by the middle controller into the tire sideslip angles of four wheels and the front wheel steering angle of the vehicle. And the latter will continue to be converted into the longitudinal forces of the front wheels by the tire inverse model. Then the two longitudinal forces of the front wheels are superimposed by the superposition scheme. That is to say, the longitudinal and lateral forces obtained by the middle controller are transformed to the longitudinal force and tire sideslip angle of each wheel required by the front wheel differential steering vehicle dynamics equation shown in Eq (4).

#### Tire inverse model

The control of tire longitudinal force can be directly realized through the control of in-wheel-motor driving or braking torque, but the control of tire lateral force cannot be directly realized. Therefore, it is necessary to use the tire inverse model to convert the tire lateral force into tire sideslip angle and the front wheel steering angle. There are two main methods to study the tire inverse model: look-up table method and analytical method. The former is to obtain the relationship table between the tire input variables and the tire force under various typical working conditions through experiments, and then find out the value of tire sideslip angle under the known conditions of tire longitudinal force, lateral force and road adhesion coefficient. Compared with the former, the tire inverse model based on the latter needs less data, which is more suitable for practical use.

The tire inverse mode adopted in this paper can be expressed as:

$$F_{yij}=-k_{i}G_{xij}\frac{\mu }{C_{ij}}{\text{tan}}^{-1}\left(\frac{C_{ij}}{\mu }\alpha_{ij}\right),\left(i=f,r,j=l,r\right),$$ (25)

where, $G_{xij}=\sqrt{1-{\left(\frac{F_{xij}}{\mu F_{zij}}\right)}^{2}}$, $C_{ij}=k_{i}\frac{\pi }{p}\frac{1}{F_{zij}}$, *p* = 2.9.

From Eq (25), the sideslip angle of each wheel for feedback to Eq (4) can be derived:

$$\alpha_{ij}=-\frac{\mu }{C_{ij}}\text{tan}\left(\frac{C_{ij}}{\mu }\frac{1}{k_{i}G_{xij}}F_{yij}\right),\left(i=f,r,j=l,r\right).$$ (26)

The steering angles of the left and right front wheels can be expressed as:

$$\delta_{fj}={\text{tan}}^{-1}\left(\frac{v_{y}+\omega l_{f}}{v_{x}-\omega l_{s}}\right)-\alpha_{fj},\left(j=l,r\right),$$ (27)

where, *δ*_*fj*_(*j* = *l*, *r*) is the steering angle of the left/right front wheel.

#### Superposition scheme of longitudinal force

Let *δ*_*f*_ = (*δ*_*fl*_ + *δ*_*fr*_)/2, and substitute it into Eq (8), the required front wheel differential driving torque Δ*T* can be obtained. According to this, the driving forces distributed to the left and right front wheels, $F_{xfl}^{\prime }$ and $F_{xfr}^{\prime }$, are as follows:

$$\left\{\begin{array}{l} F_{xfl}^{\prime }=-\frac{\Delta T}{2R_{c}}\\ F_{xfr}^{\prime }=\frac{\Delta T}{2R_{c}} \end{array}\right..$$ (28)

Let the longitudinal forces of the left and right front wheels obtained through optimization to be $F_{xfl}^{\prime \prime }$ and $F_{xfr}^{\prime \prime }$, then the actual longitudinal forces of the left and right front wheels are as follows:

$$\left\{\begin{array}{l} F_{xfl}=F_{xfl}^{\prime }+F_{xfl}^{\prime \prime }\\ F_{xfr}=F_{xrl}^{\prime }+F_{xfr}^{\prime \prime } \end{array}\right..$$ (29)

## Simulation results

In order to verify the effectiveness of the proposed controller, the double lane change maneuver is selected to conduct the comparative simulation test on the reference model, the FWDSV with hierarchical controller, and the FWDSV with SMC controller which does not consider the tire friction circle (see [27] for details). The vehicle parameters used in the simulation are shown in Table 1. When the road adhesion coefficient is 0.8, *k*_*f*_ = 95202 * 2*N*/*rad*, *k*_*r*_ = 63947 * 2*N*/*rad*. When the road adhesion coefficient is 0.2, *k*_*f*_ = 68000 * 2*N*/*rad*, *k*_*r*_ = 59000 * 2*N*/*rad*.

**Table 1:** Vehicle simulation parameters.

Parameters	Values
*b*_*e*_	100*N*
*g*	10 *m*/s^2^
*h*	054*m*
*I*_*z*_	1343*kg* · *m*^2^
*l*	0.0368*m*
*l*_*f*_	1.04*m*
*l*_*r*_	1.56*m*
*l*_*s*_	0.74*m*
*m*	1240*kg*
*r*	0.0754*m*
*R*_*c*_	0.298*m*

### High adhesion coefficient road

**Fig 5:**
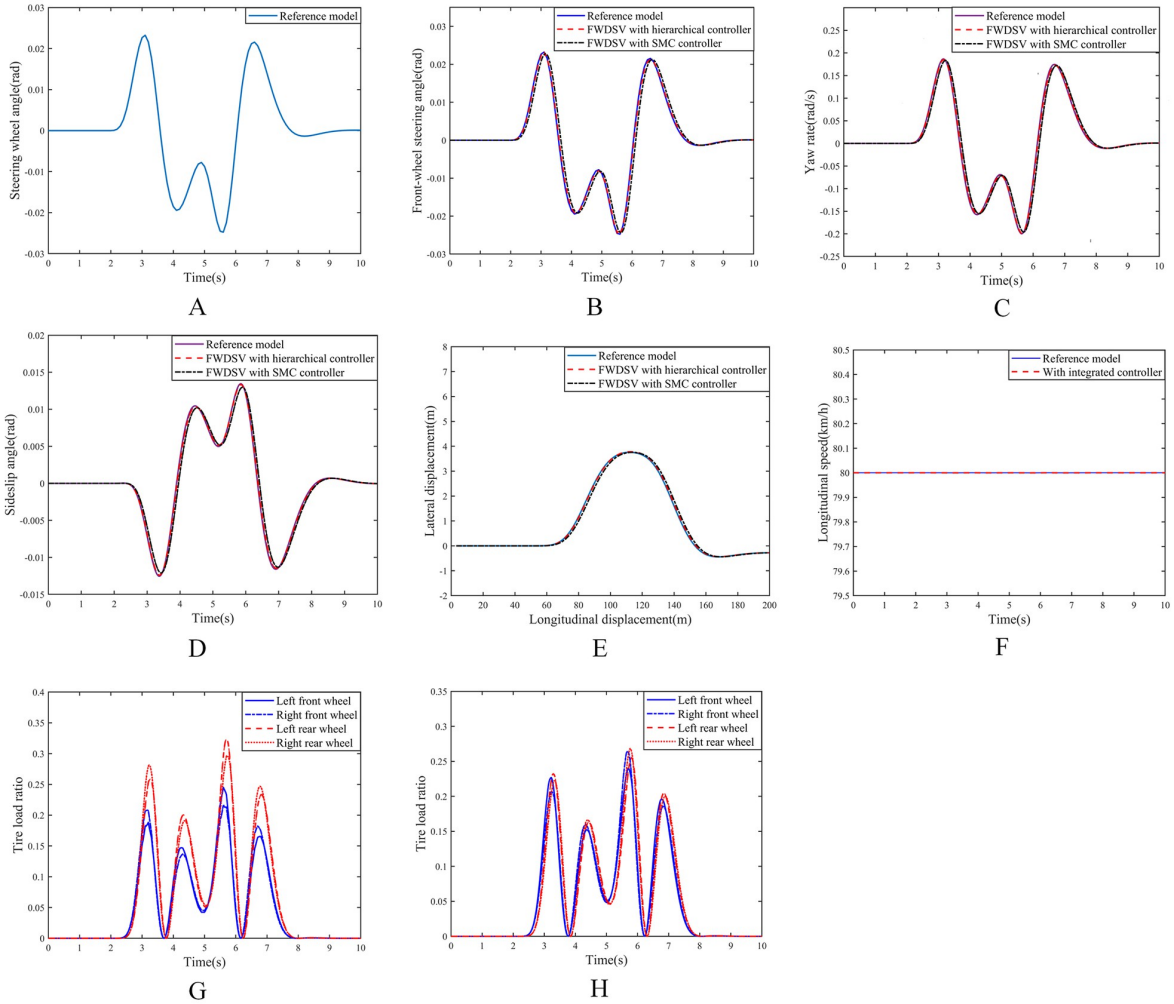
Simulation results on high adhesion coefficient road. (A) Input of steering wheel angle. (B) Curve of front wheel steering angles. (C) Curve of yaw rates. (D) Curve of sideslip angles. (E) Curve of vehicle trajectories. (F) Curve of longitudinal speeds. (G) Tire load ratio of FWDSV with hierarchical controller. (H) Tire load ratio of FWDSV with SMC controller.

Under this working condition, the road adhesion coefficient is set to 0.8, the longitudinal speeds of the reference model and the FWDSV with SMC controller are set to 80km/h, and the steering wheel angle input of the reference model is shown in Fig 5A. The front wheel steering angles, yaw rates, sideslip angles and driving trajectory curves of the three models are shown in Fig 5B–5E.

It can be seen from Fig 5B–5E that all of the front-wheel steering angles, yaw rates, sideslip angles and driving trajectories of the FWDSVs with hierarchical controller and with SMC controller can track those of the reference model very well. And their extreme values are shown in Table 2.

**Table 2:** Extreme values of response curves for three models on high adhesion coefficient road.

	Front-wheel steering angle (maximum)	Yaw rate (maximum)	Sideslip angle (minimum)	Lateral displacement (maximum)
**Reference model**	0.0232rad	0.1866rad/s	-0.0125rad	3.7581m
**FWDSV with hierarchical controller**	0.0231rad	0.1845rad/s	-0.0124rad	3.7793m
**FWDSV with SMC controller**	0.0228rad	0.1833rad/s	-0.0121rad	3.7547m

From Table 2 we can see that the extreme values of the front-wheel steering angle, the yaw rate and the sideslip angle of the FWDSV with the hierarchical controller are closer to those of the reference model than those of the FWDSV with SMC controller. However, compared to FWDSV with SMC controllers, the extreme value of the lateral displacement of the FWDSV with the hierarchical controller is slightly greater than the lateral displacement of the reference model, it is also fully acceptable because of the 4m road width.

The longitudinal speeds of the reference model and the FWDSV with hierarchical controller are shown in Fig 5F, from which the conclusion can be drawn that the longitudinal speed of the FWDSV with hierarchical controller can still be well maintained at 80km/h even when the vehicle starts to turn. The tire load ratio curves of the FWDSVs with hierarchical controller and with SMC controller are shown in Fig 5G and 5H, respectively.

It can be seen from Fig 5H that the tire load ratios of four wheels of the FWDSV with SMC controller are basically evenly distributed. However, for the FWDSV with hierarchical controller the load ratios of rear wheels are significantly greater than those of front wheels, and the tire load ratios of the left and right wheels are also obviously different, as shown in Fig 5G. The reason why the rear wheel load ratio is greater than the front wheel is that the rear wheels are mainly used for driving, and the longitudinal forces on the rear wheels have to increase to keep the constant velocity during the steering. And the reason why the left and right wheel load ratios are not equal is that the load is redistributed on the left and right wheels when the vehicle is turning.

### Low adhesion coefficient road

**Fig 6:**
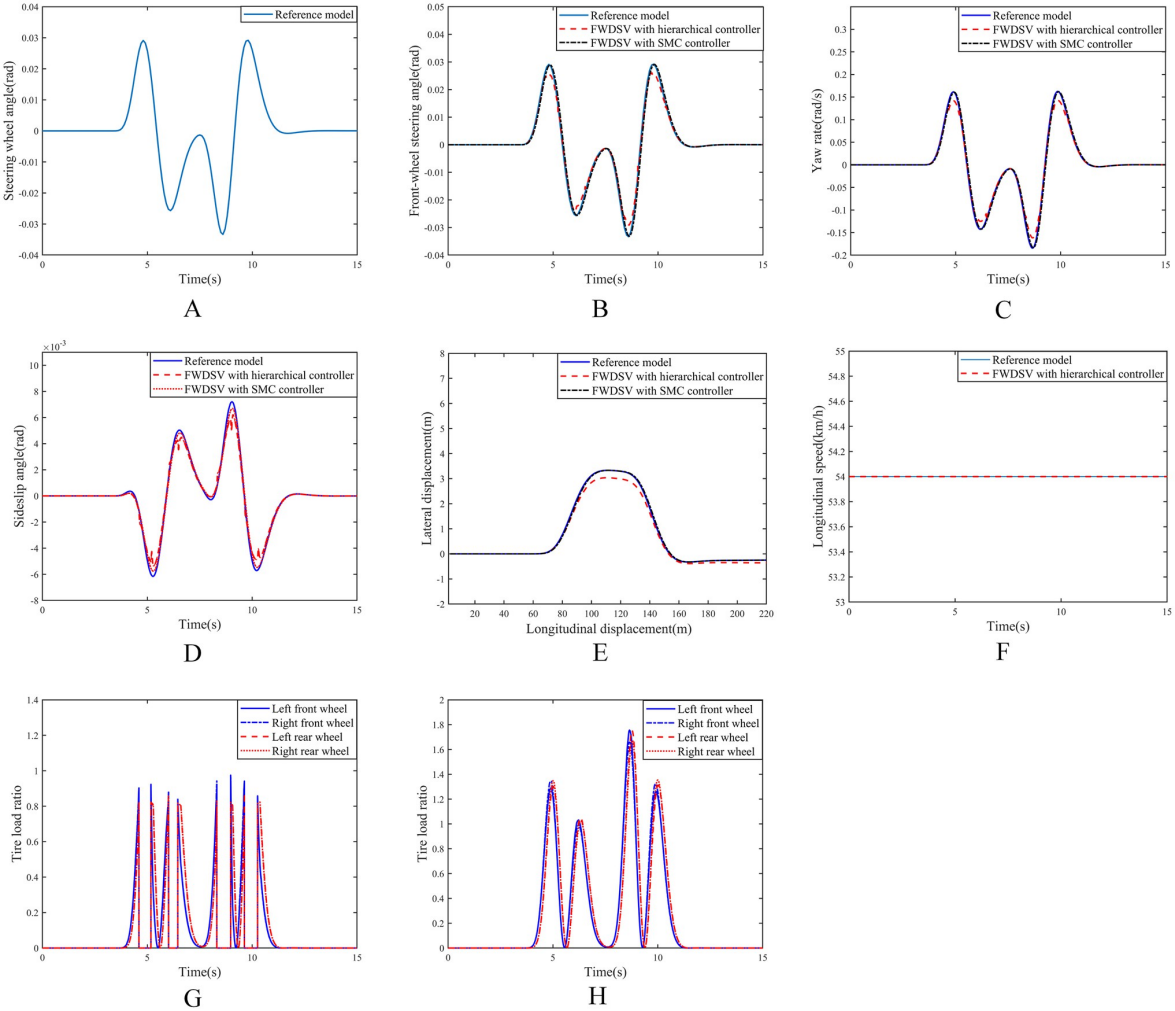
Simulation results on low adhesion coefficient road. (A) Input of steering wheel angle. (B) Curve of front wheel steering angles. (C) Curve of yaw rates. (D) Curve of sideslip angles. (E) Curve of vehicle trajectories. (F) Curve of longitudinal speeds. (G) Tire load ratio of FWDSV with hierarchical controller. (H) Tire load ratio of FWDSV with SMC controller.

Under this working condition, the vehicle speed is 54km/h, the road adhesion coefficient is 0.2, the steering wheel angle input of the reference model is shown in Fig 6A. The simulation results of the three models are shown in Fig 6B–6H.

It can be seen from Fig 6B–6E that all of the front-wheel steering angle, yaw rate, sideslip angle and driving trajectory of the FWDSVs with SMC controller can track those of the reference model very well, but those of the FWDSV with hierarchical controller are significantly different from those of the reference model. And their extreme values are shown in Table 3.

**Table 3:** Extreme values of response curves for three models on low adhesion coefficient road.

	Front-wheel steering angle (maximum)	Yaw rate (maximum)	Sideslip angle (minimum)	Lateral displacement (maximum)
**Reference model**	0.0291rad	0.1614rad/s	-0.0062rad	3.3302m
**FWDSVs with hierarchical controller**	0.0254rad	0.1414rad/s	-0.0054rad	3.0353m
**FWDSVs with SMC controller**	0.0289rad	0.1599rad/s	-0.0057rad	3.3283m

From Table 3 we can see that the extreme values of the front-wheel steering angle, the yaw rate, the sideslip angle and the lateral displacement of the FWDSV with SMC controller are closer to those of the reference model than those of the FWDSV with hierarchical controller. However, compared to FWDSV with SMC controllers, the extreme value of the lateral displacement of the FWDSV with hierarchical controller is slightly greater than the lateral displacement of the reference model, it is also fully acceptable because the road width is 4m.

The longitudinal speeds of the reference model and the FWDSV with hierarchical controller are shown in Fig 6F. It can be drawn that even on the low adhesion coefficient road the longitudinal speed of the FWDSV with hierarchical controller can still be stable at 54km/h. Fig 6G and 6H respectively show the tire load ratios of the FWDSVs with hierarchical controller and with SMC controller. It can be seen that on the low adhesion coefficient road, both of the tire load ratios are increased. However, all of the tire load ratios of the FWDSV with SMC controller exceed 1, and the maximum value even reaches 1.76 which is completely impossible in the actual situation.

## Discussion

From the analysis of Fig 5, it can be seen that on the road with high adhesion coefficient, both of the FWDSVs with hierarchical controller and with SMC controller can well track the front wheel angle, yaw rate, sideslip angle, driving trajectory and longitudinal speed of the reference model. The reason is that the road adhesion coefficient is high and the tire force is not saturated. However, the tire load ratios of the four wheels of the SMC controlled FWDSV are almost equal, and the reason why the load ratios of the rear wheels of the hierarchical controlled FWDSV are higher than those of the front wheels is that the speed of the SMC controlled FWDSV is assumed to be constant, while the hierarchical controlled FWDSV needs to realize the constant speed of the FWDSV through the driving forces of the rear wheels. In addition, the reason why the load ratios of the left and right wheels of the hierarchical controlled FWDSV are not equal is that the load is redistributed on the left and right wheels during the driving process of the vehicle. And it can be drawn that the tire load ratios of the FWDSV with hierarchical controller is more reasonably distributed according to the change of wheel vertical load, although both of the tire load ratios are not high.

**Remark 3**. Performance in all aspects of the FWDSVs with hierarchical controller and with SMC controller can well track those of the reference model on the road with high coefficient of adhesion. The reason is that the adhesion relationship between the tire and the ground can be maintained stably on high adhesion coefficient road. Although the tire load ratios of the FWDSV with hierarchical controller are different from those of the FWDSV with SMC controller, the tire load ratios of the FWDSV with SMC controller do not exceed the limit, therefore both of the vehicles can track the reference model very well.

From the analysis of Fig 6, it can be seen that the FWDSV with SMC controller can well track the front wheel angle, yaw rate, sideslip angle, driving trajectory and longitudinal speed of the reference model on the road with low coefficient of adhesion, while those of the FWDSV with hierarchical controller has some deviations from those of the reference model. This is because the FWDSV with SMC controller does not consider the tire friction circle, and the vehicle speed is assumed to be constant, and the corresponding steering is only achieved by the differential driving torque of the left and right wheels, however, the load ratio of all tires is greater than 1. This is completely inconsistent with the actual situation. Although the FWDSV with hierarchical controller has some deviations in some performance, it can ensure that all tire load ratios are less than 1 which means that this control is feasible for vehicles.

**Remark 4**. Performance in all aspects of the FWDSV with SMC controller can track those of the reference model well on low adhesion coefficient road, while those of the FWDSV with hierarchical controller have some deviations. This is because the tire friction circle is not considered for the FWDSV with SMC controller. That is to say, because the tire force saturation is not considered, each performance of the FWDSV model with SMC controller can track those of the reference model, but all of the tire load ratios are greater than 1. However, the hierarchical controller takes into account the nonlinearity of the tire and the limitations of the friction circle. Therefore, although there is a little deviation in each performance, the tire load ratios are not greater than 1. It can be drawn that the hierarchical controller proposed in this paper is effective.

## Conclusion

1. This paper focuses on how to use the over-actuation drive system to realize the differential steering for four-wheel-motor electric vehicles. The so-called differential steering refers to the steering of the vehicle through the differential driving torque between the left and right front wheels. It can not only be used as the backup system of the steering-by-wire system, but also as the single steering system of the vehicle which can make the vehicle structure simpler.
2. However, if the load of a front wheel on one side is sharply reduced due to the redistribution of the load when the vehicle is turning, the actual driving torque of the wheel will be affected which may lead to the failure of differential steering. Therefore, this paper considers the problem of tire friction circle, and designs a hierarchical control strategy to ensure that the differential steering is realized while the vehicle speed is constant.
3. Firstly, the dynamic models of the front wheel differential steering vehicle, the front wheel differential steering system and the reference vehicle are established. Then the upper controller, the middle controller and the lower controller are designed respectively.
4. Finally, simulation results show that compared with SMC controller, the FWDSV with hierarchical controller can guarantee the vehicle to track the reference model well on both of the high and low adhesion coefficient roads, and all of the tire load ratios are smaller than 1 which prove that the hierarchical controller proposed in this paper is effective.
